# The First Permanent Molar and Its Early Management

**Published:** 1884-06

**Authors:** J. Hardeman

**Affiliations:** Muscatine, Iowa.


ARTICLE III.

### THE FIRST PERMANENT MOLAR AND ITS EARLY MANAGEMENT.

BY J. HARDEMAN, D. D. S., MUSCATINE, IOWA.

This tooth, attended by so many peculiarities special to itself, when compared with the rest, furnishes cause for special diagnosis, special treatment and special prognosis. It does not differ materially from the rest, histologically or anatomically, nor radically in its functional characteristics. These therefore we pass as not strictly within the compass or importance of the question aimed at, while its more marked traits, in the view of dental practice, will furnish ample material for an earnest consideration.

We say *earnest*, as truly this is one of more than usual importance in the practice of dentistry, and of very grave interests to our patients, and the more so to both, because of the still unsettled theory and practice in regard to it, although it has been the subject of much controversy in dental assemblies and journals, and also among the leaders in the profession almost since the professor's birth.

This tooth is erupted at about the age of six or seven years, and is the first permanent tooth of the entire set as a rule. It is often regarded by the parents, and too often by the family physician, as a temporary tooth, thus leading to fatal results in its management. But we proceed to consider our subject in its more practical phrase ; one that involves the solution of the problem after these minor primary ones have passed. We are then to qualify ourselves to be fully able to bear the responsibility of deciding as experts what science, wisdom and experiences directs us to do, or how to proceed in regard to the successful management of this enigmatical tooth. This tooth comes to fill a blessed mission to the child, and if nature is not ignorantly trifled with, or too imperfectly aided, will certainly fulfil that beneficient purpose.

We will keep in view, that just at this age, when this tooth is emerging, the jaw has more teeth in it than at any other age; and more important still, more teeth in a formative state than at any other time, and too, at a period of life when the demand for element to build osseous structure the very greatest; thus aggregating an inordinate drainage of tooth making pabulum, that probably is not equaled at any other time of life. And may not this fact furnish ample cause for the great tendency of this tooth to caries? And allowing a slight digression, do not these facts admonish that the patient be nourished with food rich in tooth and osseous element? And being supplied with plenty of physical exercise, pure air, regular habits and sufficicient sunlight, etc.?

As we generally find this tooth confronting us in practice already emerged, and more or less diseased, it is more clearly our task to consider how to meet and combat the various abnormabilities threatening, and driving the patient to seek relief at our hands. If, now, we fortunately meet it before the pulp chamber has been laid open, the course of treatment is simple enough, and not likely to divide our views ; but should the devouring caries have already broken this precious precinct of vitality, then the problem presents itself, and the difficulties begin.

One says, cap the nerve and fill; another says, devitalize and fill, while a third says, do these, but if suppurated, extract; a fourth says, whenever the pulp is laid bare, extract; and a fifth says, if one is to be sacrificed, then (sound or not) all four should be extracted. Besides these many very diverse rules, others as by-rules or exceptions to the fundamental ones, are offered. This great diversity of theory must go to prove that the would-be teachers in dentistry are greatly at sea in regard to this matter, and certainly furnishes a perplexing question to the mind of the dental student.

The peculiarities attending this tooth, and giving it its special distinguishing traits, occur before majority or through adult life; and to this period of youth we propose to limit this consideration; or more definitely, we mean the period extending from the eruption of this tooth to the emerging of the dens-sapientæ.

Some very methodical authors would be likely to divide this subject into the following heads: 1. Where caries has not extended to the pulp chamber; 2. Where it has, but the pulp still remaining healthy; 3. Where the pulp is diseased; 4. Where the pulp is dead, and 5. Where the tooth is suppurated. My rule would simplify this, and present but two heads, viz: 1. When the caries has not reached the pulp chamber, and 2d. Where it has. The first may be disposed of as not likely to furnish any controversy or difference in treatment, as we would unanimously pronounce in favor of the efficiency and rectitude of filling the tooth, other things being normal. But in the second division we fully come into the arena by announcing, *we would extract.*

Whenever the pulp is exposed in this tooth at this age, and especially if the second and third molars are not missing, *treat by extraction.* To settle this in our way, we would say extensive anticipated physiological changes in adjoining parts, as well as pathological conditions of the tooth itself. must exert a large influence in treatment. The immature state of the tooth, the absolute need of pulp influence, the changes of position, in consequence of the coming second, and especially the third molars crowding for place. These go far to disqualify this tooth for usefulness and susceptibility of treatment after nerve exposure and nerve death. We think we are safe to say that where in this tooth a nerve is capped, or devitalized, or where the root membranes are suppurating, and this tooth filled at any time prior to the eruption of the dens-sapientæ, the sequel will prove a failure. Not one in fifty will remain in a useful condition at the age of twenty-five; but the rule will be deterioration that amounts to disintegration, passing generally into alveolar abscess, and the finale, quite certainly, will be the removal of the tooth.

It is hardly necessary to say that the loss of nerve influence in cutting off the further supply of mineral and other tooth elements, so much needed in this immature tooth, cannot be expected to be supplied through the peridentium. No such influence from this source can be anticipated. It absolutely requires *nerve* influence to carry onward the deposit of tooth element; and that where it is cut off there is an end to further development of this kind. The tax upon vital energy, while the struggle for position and development of the second and third molars will be too great, and pathological action is established, that generally ends the suffering by the patient's yielding to removing.

Where this tooth is removed at a period sometime before the emerging of the third molar, the second and the third will gracefully and certainly shift forwards and close up the space; securing an excellent tier of grinders, and the concentration and conservation of increased vital energy to the third molar, making that a well matured and permanent organ. While, in contradistinction, an attempt to save a first molar already in this second division, will surely entail unremunerative expense; prolongation of suffering, and approaching despair, though sometimes late, but only too sure, *the tooth is extracted after the emerging of the third molar and a permanent gap as the sure result, with the third*

*molar deficiently developed, badly crowded back and out of useful range, of but little service, and of short life.*

The statement that the early extraction of this tooth is followed by a forward dipping position of the second molar, and also of deformity by favoring malformation of the maxillary bones, etc., we can but regard as merely fanciful. The former is much oftener the result of too late or tardy extraction, when it does occur; and the latter is more generally prevented by the loss of this tooth in time, producing improved physiognomic contour, rather than damage; as, indeed, the attempt to save the one is likely to sacrifice the two.

The plan of removing the entire four first molars, where one is past salvation, in order as it is claimed, to secure and maintain perfect articulating antagonism, is, in my view, bad physiology and reprehensible practice. By close observation in hundreds of cases where but one molar of a jaw was removed, we have found nature come to the rescue, and with such complete restitution, that we can but regard the wholesale extraction of sound teeth in this case, and *a lone* for this purpose as erroneous and ill practice. And finally, my observations force me to the conclusion, that capping the nerve by any approved plan, will prove a failure in this tooth any time before the third molar is developed. And if the nerve is devitalized, no mode of root or crown filling, alone with the best therapeutic treatment, will carry the tooth even to adult age, and then worth one-half what it cost in suffering and disappointment. And if enduring perseverance has it in possession at this age. Ninety-nine chances to once, it is more or less a nuisance, and will not be tolerated but a limited period, and then its removal followed by a permanent gap, and very certainly an imperfect third molar.—*Dental Luminary*.

